# Characterization of Essential Oils from Different Taxa Belonging to the Genus *Teucrium* in Sardinia Island, Italy

**DOI:** 10.3390/plants10071359

**Published:** 2021-07-02

**Authors:** Alfredo Maccioni, Danilo Falconieri, Cinzia Sanna, Silvia Porcedda, Alessandra Piras, Andrea Maxia

**Affiliations:** 1 Co.S.Me.Se, Consorzio per lo Studio dei Metaboliti Secondari, Via Sant’Ignazio da Laconi 13, 09123 Cagliari, Italy; alfredomaccioni87@gmail.com (A.M.); a.maxia@unica.it (A.M.); 2 State Industrial Technical Institute “Michele Giua”, Via Montecassino 41, 09134 Cagliari, Italy; danilo.falconieri@tiscali.it; 3 Department of Life and Environmental Sciences, University of Cagliari, Via S. Ignazio da Laconi 13, 09123 Cagliari, Italy; 4 Department of Chemical and Geological Sciences, University of Cagliari, Cittadella Universitaria, S.S. 554, 09042 Monserrato, Italy; porcedda@unica.it (S.P.); apiras@unica.it (A.P.)

**Keywords:** *Teucrium*, Lamiaceae, Sardinia, essential oil, GC–FID, GC–MS, monoterpenes, sesquiterpenes, diterpenes, phenylpropanoids compounds

## Abstract

The genus *Teucrium* L. (Lamiaceae) is a genus growing in mild climate zones, particularly in the Mediterranean Basin and Central Asia. It is represented by 11 taxa in Sardinia (Italy), living commonly in sunny habitats. In this study, the following eight Sardinian *Teucrium* taxa were selected, and the essential oils (EOs), obtained by stem distillation, were analyzed by GC–FID and GC–MS: *T. capitatum* subsp. *capitatum*, T. *chamaedrys* subsp. *chamaedrys*, *T. flavum* subsp. *glaucum*, *T*. *marum*, *T. massiliense*, *T. scordium* subsp. *scordioides*, *T. scorodonia*, and *T. subspinosum*. The comprehensive analyses led to the identification of 87 constituents representing the majority of the volatile compounds. Significant differences, both qualitative and quantitative, were observed between the taxa. Overall, monoterpenes and sesquiterpenes characterized all *Teucrium* EOs: *T. capitatum* subsp. *capitatum* and *T. flavum* subsp. *glaucum* revealed the highest content of monoterpene hydrocarbons, while in the other *Teucrium* taxa sesquiterpene hydrocarbons prevailed. Worthy of note, diterpenes were found only in *T*. *marum* and *T. subspinosum*, whereas *T. massiliense* was rich in non-terpenic oxygenated compounds. To the best of our knowledge, this is the first comprehensive report on the chemical composition of EOs obtained from Sardinian *Teucrium* species.

## 1. Introduction

The genus *Teucrium* (germander) includes 300 taxa belonging to the Lamiaceae family, distributed in Europe, North Africa, and temperate areas of Asia, but mainly concentrated in the Mediterranean region [1,2]. It is represented by herbs or shrubs, with tubular or campanulate calyx, 2-lipped or actinomorphic, 5-toothed, the teeth equal or the upper larger; corolla with one 5-lobed lip; tube without a ring of hairs inside, often included in the calyx; and nutlets smooth or reticulate. Many taxa of the genus *Teucrium* have been used in traditional medicine as stimulants, tonics, and stomach-ache remedies, for their antioxidant, antifungal, antibacterial, antidiabetic, antiseptic, astringent, antispasmodic, anti-inflammatory, antianemic, and cicatrizant properties, and to treat skin diseases [3,4,5,6,7]. Phytochemical studies reported the presence of both volatile and non-volatile compounds. Of the latter, several neo-clerodane diterpenoids were isolated and considered as chemotaxonomic markers of the genus [4,8,9,10], along with iridoids, sesquiterpenes, triterpenes, abietane diterpenes, flavonoids, and other classes of specialized metabolites [11]. Much scientific research has already been carried out on essential oils (EOs), since 99 *Teucrium* taxa have been investigated to date [7], highlighting the main volatile compounds, which are generally sesquiterpene hydrocarbons. The interest in studying this genus lies also in its documented in vitro and in vivo biological activities, such as antimicrobial, antioxidant, anti-inflammatory, anticancer, antinociceptive, and antifungal properties [4,7,12,13]. Moreover, phytotoxic, insecticidal, and antileishmanial effects have also been reported for several species, especially for *T. polium* [14,15,16]. The genus *Teucrium* is represented by 21 taxa in Italy, 11 of which grow wild in Sardinia (Italy), as reported by Bartolucci et al. [17]. Sardinia, with an area of 24.089 km^2^, is the second island of the Mediterranean Basin. Thanks to its 2441 taxa of native vascular plants [17] of which ca. 12% are endemic of the Sardinian–Corsican biogeographic province [18], it represents one of the Mediterranean biodiversity hotspots [19,20,21]. This endemic richness is attributable to the long geological history and geographical isolation, in addition to the high geomorphological diversity [21,22,23], and it could represent a great source of phytoconstituents, often unique and structurally diverse, due to adaptation to the local environment [24]. Out of Sardinian *Teucrium* taxa, the following eight native and spontaneous taxa were selected in this study: *T. capitatum* L. subsp. *capitatum* (felty germander), *T. chamaedrys* L. subsp. *chamaedrys* (wall germander), *T. flavum* L. subsp. *glaucum* (Jord. and Fourr.) Ronniger (yellow germander), *T. marum* L. (cat thyme), T. *massiliense* L. (Marseille germander), *T. scordium* L. subsp. *scordioides* (Schreb.) Arcang. (water germander), *T. scorodonia* L. (wood sage/woodland germander), and *T. subspinosum* Pourr. ex Willd (spiny germander). The three remaining species, *T. montanum* L. (mountain germander), *T. fruticans* L. subsp. *fruticans*, and *T. flavum* L. subsp. *flavum*, were not studied. The former was not selected because of its rareness in the island: in fact, it is exclusive to a very limited area in the calcareous mountains [25]; *T. fruticans* subsp. *fruticans* is not native and spontaneous in Sardinia, therefore it is considered to be an alien species [17]; the latter, common in the Italian peninsula, was considered replaced in Sardinia by *T. flavum* subsp. *glaucum* [25,26,27,28]. In Sardinian ethnobotany, the species selected in our study, except for *T. subspinosum* and *T. scorodonia*, have been reported to treat several diseases, confirming the wide use of the genus *Teucrium* in the traditional medicine systems all over the world. *T. capitatum* subsp. *capitatum* has been used to treat gastralgia, menopause-associated disorders, cold, and as cicatrizant [29]; *T. chamaedrys* subsp. *chamaedrys* as febrifuge, antimalaric, analgesic, anti-catarrhal, and depurative [29]; *T. flavum* subsp. *glaucum* as cicatrizant, antiseptic, febrifuge, antirheumatic, and to treat sciatica and dislocation [29]; *T. marum* has been reported as antibacterial, antifungal, febrifuge, cicatrizant, antispasmodic, analgesic, antirheumatic, anti-catarrhal, and to treat respiratory diseases [3,29]; *T. massiliense* as antimalaric, febrifuge, and cicatrizant [29,30]; *T. scordium* subsp. *scordioides* as antiseptic and anthelmintic [29].

The aim of this research is to provide, for the first time, a comprehensive study on EOs composition of eight wild *Teucrium* taxa collected in Sardinia, and to compare our data with the available literature.

## 2. Results and Discussion

**Figure 1:**
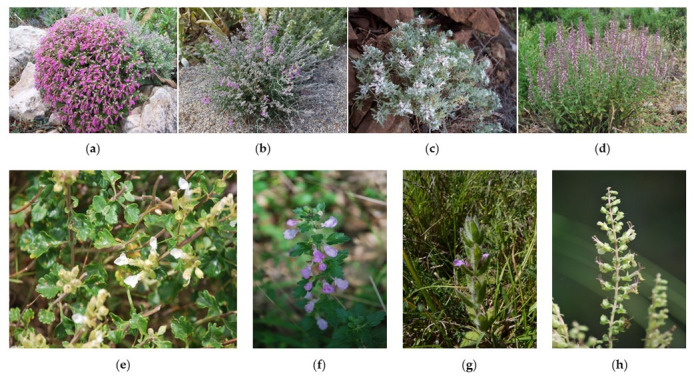
*Teucrium* taxa selected for this study: (**a**) *T. marum*; (**b**) *T. subspinosum*; (**c**) *T. capitatum* subsp. *capitatum*; (**d**) *T. massiliense*; (**e**) *T. flavum* subsp. *glaucum*; (**f**) *T. chamaedrys* subsp. *chamaedrys*; (**g**) *T. scordium* subsp. *scordioides*; (**h**) *T. scorodonia*.

The aim of our study was to investigate the volatile fraction obtained from eight Sardinian *Teucrium* taxa (Figure 1), to improve the knowledge on this genus. In fact, even though the *Teucrium* genus has been largely studied all over the world, especially for the chemical composition of the essential oils [7], to our knowledge, a comprehensive analysis focusing on Sardinian taxa has never been provided. Furthermore, a comparison between our results and literature data was performed. Many *Teucrium* species exhibited interesting biological properties such as antioxidant [31], anti-inflammatory [32,33], antitumor [32,33,34], and antimicrobial [5,31], justifying the widespread applications in the ethnomedicine of several countries. A comprehensive survey of traditional uses, chemical composition, and biological properties of the essential oils isolated from *Teucrium* taxa has been recently published [7].

In our study, all samples were collected during the full flowering period (June–July). Even though the harvesting time is species-specific, it has been previously reported that flowering stage is the best time to harvest aromatic plants belonging to the Lamiaceae family [35,36]. It is known that the phenological stage can greatly affect essential oil composition [37]. In fact, it has been observed that the amount of sesquiterpenes, compounds usually dominant in *Teucrium* species, slightly decreased in the budding stage, counterbalanced by an increase in oxygenated compounds [38]. Furthermore, the biosynthetic pathway for terpenoids is heavily dependent on environmental factors, such as temperature, water availability, soil composition, and altitude [38,39,40,41,42]. Information on local climate conditions, substratum, altitude, type of habitat, bioclimate–isobioclimates, type of vegetation, and vegetation series of collection sites is provided in Table S1. Except for *T. chamaedrys* subsp. *chamaedrys* and *T. scordium* subsp. *scordioides,* which were collected from the same location, the other taxa were harvested in several sites located at altitudes between 10 m and 803 above sea level.

Overall, 87 compounds were identified (Table 1), covering a percentage of the total EOs ranging from 85.31% (*T. marum*) to 99.8% (*T. capitatum* subsp. *capitatum*).

The phytochemical analyses showed that, except for *T. capitatum* subsp. *capitatum* and *T. flavum* subsp. *glaucum*, sesquiterpenes hydrocarbons were the main fraction of the EOs, accounting for 45.18–92.0% of the whole samples. Differently, *T. capitatum* subsp. *capitatum* and *T. flavum* subsp. *glaucum* EOs were dominated by monoterpene hydrocarbons (87.63 and 76.85%, respectively). Interestingly, diterpene hydrocarbons and oxygenated phenylpropanoids were identified only in *T. marum* and *T. subspinosum*, in confirmation of their similarity, both morphological and phytochemical. Moreover, *T. massiliense* was rich in non-terpenic oxygenated compounds (36.02%).

As shown in Table 1, the main components detected in EO obtained from *Teucrium marum* are β-bisabolene (23.04%), β-sesquiphellandrene (17.78%), 3E-cembrene A (14.01%), and (E)-caryophyllene (9.08%). This species, known as “cat thyme” or “mint plant”, was previously studied revealing a high variability in the EO composition. Samples originated from Balearic Island were characterized by a high percentage of dolichodial, an iridoid identified in both the organic solvent extracts and simultaneous distillation–extraction [43,44]. This compound exerts insecticidal activity and may represent a plant defensive agent [45]. It was also detected in emitted volatile fractions from Corsican samples [5,46], as well as in previously analyzed Sardinian samples [31,43], but not in our oil, pointing out that environmental factors strongly influence the chemical composition of EO, as widely reported in literature [47,48]. Indeed, our samples were collected at sea level, whereas Ricci et al. [31] investigated aerial parts harvested at 800 m altitude. Interestingly, in our study *T. marum* EO evidenced a similar pattern to *Teucrium subspinosum*, although quantitative differences in the main compounds have been highlighted. These two taxa are often confused and their taxonomic rank and distribution are still open to discussion [28]. Recently, this taxon was described as endemic to south-western Sardinia and Majorca [25]. Our results, as well as previous phytochemical reports analyzing the EOs from *T. marum* and *T. subspinosum* gathered in Sardinia and the Balearic Islands [43,44], were not conclusive and they corroborated the remarkable affinity between the two entities. Differently from what was observed in the volatile fraction, the ethanol extract showed significant differences in the phenylpropanoid glycosides content, suggesting these compounds as markers of these taxa [4]. In fact, verbascoside was detected in both species, but arabinosyl-verbascoside appeared not to be present in *T. marum*. Both species exhibited antioxidant activity in the DPPH test [4].

Regarding *Teucrium massiliense*, our results confirmed a very high variability. The main compounds detected in the EO, according to previous reports [5,49] were 6-methyl-3-heptyl acetate (23.55%), followed by γ-muurolene (10.97%), (E)-β-farnesene (8.44%), ar-curcumene (7.56%), 3-octenyl acetate (7.33%), α-zingibergene (6.37%), and (Z)-α-bisabolene (5.85%). Djabou et al. [49] reported the 6-methyl-3-heptyl acetate for the first time as a natural compound in Corsican and Sardinian oil obtained from *T. massiliense*. Excepting for the high amount of this ester, our results significantly differed from the Sardinian and Corsican EOs reported in the literature [5,49,50]. For instance, our EO was lacking in germacrene D, a volatile compound detected in all samples previously analyzed. *T. massiliense* has been studied for its biological activities, and antioxidant, antifungal and antibacterial effects have been documented [5,50], suggesting its potential use in food preservatives and to prevent the growth of nosocomial bacteria.

*Teucrium capitatum* subsp. *capitatum* belongs to the *Polium* section, a complex section with considerable morphological variations between populations. Previously known as *T. polium* subsp. *capitatum*, it has been largely studied all over the Mediterranean Basin, evidencing a very high polymorphism and variability in its volatile constituents. According to a recent report [51], our EO consisted essentially of monoterpene hydrocarbons, being rich in limonene (30.35%), α-pinene (29.79%), β-pinene (9.96%), and myrcene (9.63%). To the best of our knowledge, there is only one report on the EO composition of *T. capitatum* subsp. *capitatum* originated from Sardinia [13]. It highlighted the existence of two chemotypes: one characterized by limonene/α-pinene/(E)-nerolidol, predominant in plants growing at sea level, and a second rich in limonene/α-pinene/α-trans-bergamotene/humulene epoxide II, found in mountain samples. Our EO, obtained from aerial parts collected in a costal garrigue, lacked in α-trans-bergamotene, humulene epoxide II, and (E)-nerolidol, confirming the very high variability of this taxon, in accordance with several reports [5,14,32,52,53,54]. Differently from our EO, in which limonene was the main compound (30.35%), all EOs obtained from Corsican *T. capitatum* subsp. *capitatum* were characterized by a lower amount of this monoterpene hydrocarbon, ranging from 3.0 to 5.2%. In addition, a greater quantity of α-thujene (5–8.1%) distinguishes Corsican samples [5,52,53]. We did not detect in our EO the germacrene D, a compound abundant in samples originated from Greece [54,55], Balkan Peninsula [56], and Algeria [57]. Finally, samples from Algeria and Portugal were rich in *r*-cadinol, differently from all the other analyzed EOs [57,58]. *T. capitatum* subsp. *capitatum* was recently reported for its antifungal activity, particularly against dermatophytes and *Cryptococcus neoformans,* and able to inhibit *Candida albicans* germ tube formation [13]. It also showed anti-inflammatory and antiproliferative effects on lung carcinoma, colorectal adenocarcinoma, and amelanotic melanoma cell lines, even though the mechanism of action has not yet been explained [32].

*Teucrium flavum* subsp. *glaucum* grows spontaneous in Sardinia, Calabria, Puglia [17] and Corsica. To the best of our knowledge, there are only two reports on this taxon originated from Corsica [5,59]. The majority of scientific researches concerns the other subspecies, namely *T. flavum* subsp. *flavum*, which is more common [5,27,59,60,61,62]. Both subspecies exhibited morphological differences in the plant size, leaf length, and the presence of peltate hairs [1]. In addition, chemical and genetic differences between them have been reported in Corsica [5]. Our EO was rich in limonene (26.96%), followed by α-pinene (25.07%), β-pinene (15.12%), (Z)-β-ocimene (8.32%) and γ-muurolene (5.77%), in accordance with what was observed in Corsican samples [5,59]. Similarly to *T. capitatum* subsp. *capitatum,* the EO of *Teucrium flavum* subsp. *glaucum* exhibited anti-inflammatory and cytotoxic activities against three tumor cell lines [32]. Recently, from the ethyl acetate extract obtained from Sardinian samples, several compounds with inhibitory activity towards the HIV-1 reverse transcriptase-associated RNase H function have been isolated [63].

*Teucrium scordium* subsp. *scordioides* is a perennial herb distributed in wet soil and swamps of southern and south-eastern Europe, Asia Minor, and Morocco. With regard to the analysis of volatile fraction, only two reports have been published concerning Serbian and Sicilian populations [64,65]. Our EO was characterized by a high amount of germacrene D (25.05%), followed by δ-cadinene (12.85%), allo-aromadendrene (11.25%), α-cadinol (6.15%), and germacrene D-4-ol (5.95%), showing significant differences with respect to the ones obtained from Sicilian and Serbian samples. In fact, the EO obtained from Sicilian *T. scordium* subsp. *scordioides* was characterized by the presence of caryophyllene oxide (25.8%), α-pinene (19.4%), and β-pinene (8.5%), while the Serbian one contained menthofuran as the main component, a compound totally absent in the other accessions.

*Teucrium chamaedrys* subsp. *chamaedrys* grows wild in Central Europe, Asia, and North Africa. This plant is common in Corsican and Sardinian islands. It has been largely studied, both for its volatile and non-volatile fractions. Neo-clerodane diterpenoid derivatives, along with phenylpropanoids and steroidal compounds have been isolated from its aerial parts [66,67,68,69]. There have been previous studies in relation to the EO and quantitative differences in the number of major components have been reported. Indeed, whereas samples collected in Corsica and Sardinia and studied by Muselli et al. [70] were characterized by a high amount of (E)-β-caryophyllene, as well as Serbian samples [71], our EO was dominated by germacrene D (41.85%), similarly to the Turkish ones [72,73]. In our EO, (E)-caryophyllene (29.25%), α-humulene (6.98%) and (E)-β-farnesene (6.20%) were also identified in high amounts.

*Teucrium scorodonia* EO was characterized by the highest percentage of sesquiterpene hydrocarbons (92.2%) among the taxa studied. The main components were (E)-caryophyllene (18.95%), α-cubebene (14.51%), germacrene B (14.05%), β-cubebene (8.51%), α-humulene (7.46%), germacrene D (7.35%), and α-gurjunene (5.57%). To the best of our knowledge, there are few reports on this species, concerning samples collected in Italy, Spain, and Corsica [74,75,76,77]. These articles revealed a high variability in the volatile components, especially depending on the part of plant analyzed.

## 3. Materials and Methods

### 3.1. Plant Material and Essential Oil

Flowering aerial parts of each taxon selected were collected randomly from wild populations, from June to July 2015, in different Sardinian localities. Species were botanically identified by Dr. Alfredo Maccioni and voucher specimens were deposited at the General Herbarium of the Department of Life and Environmental Sciences, University of Cagliari (Herbarium CAG). The name of collection sites, along with voucher specimens, GPS coordinates, local climate, and environmental conditions are listed in Table S1.

Plant material was dried at 40 °C to constant weight and then subjected to steam distillation in order to obtain EOs, according to the European Pharmacopoeia, 10th edition. EOs were stored at 4 °C in the dark until the chemical analyses.

### 3.2. GC-FID and GC-MS Analysis

Quantitative analyses were performed on an Agilent 7890A GC (3 repetitions of each sample) equipped with a flame ionization detector (FID) and a 30 m × 0.25 mm i.d. with a 0.25 μm stationary film thickness HP-5 capillary column [78]. Quantification of constituents was calculated by integration of GC-FID peak areas without using the response correction factors. Qualitative analyses were carried out in a gas chromatograph (Agilent 6890N) equipped with a 30 m × 0.25 mm i.d. with a 0.25 μm stationary film thickness HP-5 ms capillary column (Agilent J&W), coupled with a mass selective detector with an electron ionization device (EI) and a quadrupole analyzer (Agilent 5973) [78]. The compounds of the samples were identified by comparing mass spectra fragmentation patterns with those of a computer library, and linear retention indices (RI) were based on a series of C_8_-C_26_ n-alkanes homologous to those reported in the literature [79].

## 4. Conclusions

This study is a comprehensive report on the EO compositions of eight wild *Teucrium* taxa, with the aim to increase the knowledge of the genus *Teucrium* in Sardinia (Italy). In fact, along with species largely investigated, such as *T. chamaedrys* subsp. *chamaedrys*, *T. marum*, *T. massiliense*, and *T. capitatum* subsp. *capitatum*, we analyzed, for the first time, the EOs obtained from some species not yet studied in the island (*T. scorodonia*, *T. scordium* subsp. *scordioides*, *Teucrium flavum* subsp. *glaucum*, and *T. subspinosum*). These species, except for *T. subspinosum* and *T. scorodonia*, were reported in Sardinian ethnobotany, confirming the wide use of the genus *Teucrium* in the traditional medicine systems all over the world. The EOs of each taxon were characterized by GC–FID and GC–MS, and the differences with respect to the literature highlighted. Our results showed, according to the available data, a very high variability of this genus, since significant differences, both qualitative and quantitative were found in the volatile fraction. This variability could be to the consequence of genetic, seasonal, and environmental factors, as is widely documented in literature. Overall, 87 constituents were detected and, except for *T. chamaedrys* subsp. *chamaedrys* and *T. flavum* subsp. *glaucum*, both characterized by a large amount of monoterpene hydrocarbons, sesquiterpenes hydrocarbons were the main fraction in all the other taxa. Worthy of note, diterpene hydrocarbons and oxygenated phenylpropanoids were identified only in *T. marum* and *T. subspinosum*, highlighting their similarity, both morphological and phytochemical. Moreover, *T. marum* was rich in non-terpenic oxygenated compounds.

## Figures and Tables

**Table 1:** List of the components identified in essential oils obtained from eight Sardinian *Teucrium* taxa selected in this study. For each compound, the relative quantitative amount (area %), expressed as the mean of three analytical repetitions ± SD, is reported. Tmar (*T. marum*), Tsub (*T. subspinosum*), Tmas (*T. massiliense*), Tcsc (*T. capitatum* subsp. *capitatum*), Tfsg (*T. flavum* subsp. *glaucum*), Tsss (*T. scordium* subsp. *scordioides*), Tcha (*T. chamaedrys* subsp. *chamaedrys*), and Tsco (*T*. *scorodonia*).

RI ^a^	Compound	Tmar	Tsub	Tmas	Tcsc	Tfsg	Tsss	Tcha	Tsco
930	α-thujene	-	-	-	1.02 ± 0.10	-	-	-	-
938	α -pinene	-	-	-	29.79 ± 0.20	25.07 ± 0.18	4.81 ± 0.05	1.82 ± 0.02	-
952	camphene	-	-	-	0.35 ± 0.00	-	-	-	-
958	thuja-2,4(10)-diene	-	-	-	0.65 ± 0.01	-	-	-	-
976	sabinene	-	-	-	1.39 ± 0.01	-	-	-	-
980	β-pinene	-	-	-	9.96 ± 0.03	15.12 ± 0.04	2.75 ± 0.04	0.92 ± 0.00	-
992	myrcene	-	-	-	9.63 ± 0.02	1.38 ± 0.05	-	-	-
1019	iso-sylvestrene	-	-	-	0.29 ± 0.00	-	-	-	-
1027	o-cymene	-	-	-	1.16 ± 0.02	-	-	-	-
1032	limonene	-	-	-	30.35 ± 0.05	26.96 ± 0.06	0.79 ± 0.01	0.58 ± 0.01	-
1040	(Z)-β-ocimene	-	-	1.75 ± 0.04	-	8.32 ± 0.04	1.95 ± 0.03	-	-
1051	(E)-β-ocimene	-	-	-	1.98 ± 0.00	-	-	-	-
1062	γ-terpinene	-	-	-	0.52 ± 0.01	-	-	-	-
1089	6-methyl-3-heptyl acetate	-	-	23.55 ± 0.20	-	-	-	-	-
1090	terpinolene	-	-	-	0.54 ± 0.01	-	-	-	-
1100	linalool	-	-	2.79 ± 0.04	-	-	-	-	-
1105	2-methyl butyl-2-methyl butyrate	-	0.42 ± 0.00	4.28 ± 0.05	-	-	-	-	-
1109	2-methyl butyl isovalerate	-	-	0.86 ± 0.17	-	-	-	-	-
1113	1-octen-3-yl acetate	-	0.37 ± 0.01	-	-	-	-	-	-
1126	3-octenyl acetate	-	-	7.33 ± 0.05	-	-	-	-	-
1128	α-campholenal	-	-	-	1.07 ± 0.02	-	-	-	-
1141	trans-pinocarveol	-	-	-	0.58 ± 0.01	-	-	-	-
1147	trans-verbenol	-	-	-	0.52 ± 0.01	-	-	-	-
1156	citronellal	-	0.41 ± 0.03	-	-	-	-	-	-
1164	pinocarvone	-	-	-	0.60 ± 0.01	-	-	-	-
1195	myrtenal	-	-	-	1.08 ± 0.12	-	-	-	-
1198	estragole	1.12 ± 0.01	4.95 ± 0.02	-	-	-	-	-	-
1231	citronellol	-	0.52 ± 0.01	-	-	-	-	-	-
1241	pulegone	-	-	-	-	-	-	0.61 ± 0.00	-
1245	carvone	-	-	-	0.72 ± 0.05	-	-	-	-
1286	isobornyl acetate	-	-	-	1.08 ± 0.01	2.25 ± 0.03	-	-	-
1327	myrtenyl acetate	-	-	-	0.90 ± 0.01	-	-	-	-
1352	α-cubebene	-	-	-	-	-	-	-	14.51 ± 0.06
1356	cytronellyl acetate	-	2.46 ± 0.02	1.57 ± 0.01	-	-	-	-	-
1372	α-ylangene	-	-	-	-	-	-	-	0.61 ± 0.01
1376	α-copaene	-	-	-	-	-	2.48 ± 0.01	-	4.49 ± 0.02
1384	β-bourbonene	-	-	1.75 ± 0.10	-	-	1.02 ± 0.01	1.16 ± 0.01	-
1385	geranyl acetate	3.13 ± 0.07	2.75 ± 0.13	-	-	-	-	-	-
1390	β-cubebene	-	-	-	-	-	0.90 ± 0.00	-	8.51 ± 0.03
1409	α-gurjunene	-	-	-	-	-	-	-	5.57 ± 0.03
1420	(E)-caryophyllene	9.08 ± 0.03	22.55 ± 0.06	-	3.06 ± 0.07	-	2.96 ± 0.00	29.25 ± 0.04	18.95 ± 0.03
1437	α-trans-bergamotene	4.38 ± 0.03	2.25 ± 0.01	-	-	-	0.41 ± 0.00	-	-
1439	α-guaiene	-	-	-	-	-	-	-	0.67 ± 0.00
1453	α-humulene	1.97 ± 0.02	5.95 ± 0.01	-	-	-	-	6.98 ± 0.01	7.46 ± 0.02
1459	(E)-β-farnesene	1.49 ± 0.01	0.70 ± 0.01	8.44 ± 0.01	-	3.04 ± 0.03	-	6.20 ± 0.01	-
1460	allo-aromadendrene	-	-	-	-	-	11.25 ± 0.03	-	0.34 ± 0.00
1480	γ-muurolene	-	0.95 ± 0.00	10.97 ± 0.02	-	5.77 ± 0.09	0.66 ± 0.01	-	-
1481	germacrene D	-	-	-	-	-	25.05 ± 0.07	41.85 ± 0.09	7.35 ± 0.03
1483	ar-curcumene	-	-	7.56 ± 0.01	-	-	-	-	-
1487	aristolochene	-	-	-	-	-	-	-	0.74 ± 0.00
1490	β-selinene	-	-	-	0.62 ± 0.02	-	-	-	0.63 ± 0.00
1493	trans-muurola-4(14),5-diene	-	-	-	-	-	0.53 ± 0.01	-	0.72 ±0.01
1496	α-zingiberene	0.78 ± 0.01	0.74 ± 0.00	6.37 ± 0.02	-	-	-	-	1.32 ± 0.01
1499	bicyclogermacrene	-	-	-	-	-	1.68 ± 0.01	2.63 ± 0.13	0.34 ± 0.01
1499	α-muurolene	-	-	-	-	-	1.95 ± 0.01	-	-
1504	γ-patchoulene	-	-	-	0.70 ± 0.03	-	-	-	0.59 ± 0.00
1508	(Z)-α-bisabolene	-	-	5.85 ± 0.01	-	2.12 ± 0.05	0.50 ± 0.01	-	1.25 ± 0.01
1509	β-bisabolene	23.04 ± 0.08	19.85 ± 0.02	-	-	-	-	-	-
1514	γ-cadinene	-	-	-	-	-	4.65 ± 0.02	0.34 ± 0.01	0.90 ± 0.01
1523	δ-cadinene	-	-	-	0.79 ± 0.02	-	12.85 ± 0.08	2.35 ± 0.01	2.72 ± 0.00
1525	β-sesquiphellandrene	17.78 ± 0.05	12.85 ± 0.03	4.24 ± 0.01	-	-	-	-	-
1528	zonarene	-	-	-	-	-	-	-	0.28 ± 0.00
1537	α-cadinene	-	-	-	-	-	0.40 ± 0.01	-	-
1544	elemol	-	-	-	-	-	-	-	1.65 ± 0.09
1557	germacrene B	-	-	-	-	-	-	-	14.05 ± 0.04
1565	(E)-nerolidol	-	0.29 ± 0.01	-	-	-	-	-	-
1575	germacrene D-4-ol	-	-	-	-	-	5.95 ± 0.02	0.50 ± 0.01	-
1576	spathulenol	-	-	1.70 ± 0.01	-	-	-	-	-
1581	caryophyllene oxide	1.89 ± 0.01	7.56 ± 0.01	-	0.45 ± 0.01	-	-	1.29 ± 0.01	1.65 ± 0.00
1600	ledol	-	-	-	-	-	0.48 ± 0.01	-	0.48 ± 0.00
1606	humulene epoxide II	-	1.58 ± 0.00	-	-	-	-	-	0.39 ± 0.00
1627	1-epi-cubenol	-	0.30 ± 0.01	-	-	-	0.52 ± 0.00	-	0.22 ± 0.00
1634	caryophylla-4(12),8(13)-dien-5α-ol	-	0.45 ± 0.00	-	-	-	-	-	-
1641	epi-α-cadinol	-	-	-	-	-	4.65 ± 0.03	0.74 ± 0.06	0.62 ± 0.02
1645	α-muurolol	-	-	-	-	-	0.75 ± 0.01	-	-
1648	β-eudesmol	-	-	-	-	-	2.27 ± 0.02	-	-
1653	α-cadinol	-	-	-	-	-	6.15 ± 0.05	0.84 ± 0.01	0.62 ± 0.05
1662	(E)-bisabol-11-ol	0.59 ± 0.03	-	-	-	-		-	-
1670	(Z)-α-santalol	-	0.95 ± 0.01	-	-	-	-	-	-
1678	helifolenol A	0.67 ± 0.02	-	-	-	-	-	-	-
1683	α-bisabolol	1.08 ± 0.03	0.88 ± 0.00	-	-	-	-	-	-
1687	shyobunol	-	-	-	-	-	0.62 ± 0.01	-	-
1688	(Z)-apritone	0.98 ± 0.02	-	-	-	-	-	-	-
1696	β-acorenone	0.87 ± 0.02	-	-	-	-	-	-	-
1831	(Z)-nuciferol acetate	-	-	1.54 ± 0.01	-	-	-	-	-
1953	3E-cembrene A	14.01 ± 0.12	4.70 ± 0.01	-	-	-	-	-	-
1968	3Z-cembrene A	2.45 ± 0.03	0.45 ± 0.01	-	-	-	-	-	-
**Total identified**		85.31	94.88	90.55	99.8	90.03	98.98	98.06	97.63
**Monoterpene Hydrocarbons**		-	-	1.75	87.63	76.85	10.3	3.32	-
**Oxygenated Monoterpenes**		3.13	6.14	4.36	6.55	2.25	-	0.61	-
**Sesquiterpene Hydrocarbons**		58.52	65.84	45.18	5.17	10.93	67.29	90.76	92.0
**Oxygenated Sesquiterpenes**		6.08	12.01	3.24	0.45	-	21.39	3.37	5.63
**Diterpene Hydrocarbons**		16.46	5.15	-	-	-	-	-	-
**Oxygenated Phenylpropanoids**		1.12	4.95	-	-	-	-	-	-
**Non-terpenic oxygenated compounds**		-	0.79	36.02	-	-	-	-	-

^a^ Linear retention index on a HP-5ms capillary column.

## Data Availability

All data are presented in this report.

## Supplementary Materials

The following are available online at https://www.mdpi.com/article/10.3390/plants10071359/s1, Table S1: List of Sardinian *Teucrium* taxa selected for this study.
