# Supplementary material for: Characterization of Essential Oils from Different Taxa Belonging to the Genus Teucrium in Sardinia Island, Italy
Source: Plants (Basel). 2021 Jul 2;10(7):1359. doi: 10.3390/plants10071359 (PMC8309330; doi:10.3390/plants10071359)
Supplement: Supplementary file 1 [file plants-10-01359-s001.zip › plants-1267399-supplementary.pdf]

Table S1. List of Sardinian *Teucrium* taxa selected for this study

Taxon	Label	Voucher specimen	Collection sites	GPS coordinates	Information on collection sites
Teucrium capitatum L. subsp. *capitatum*	Tcsc	CAG 1123a	Su Crastu biancu - San Vero Milis (OR)	40° 2'48.97''N 8°25'51.09''E	(1) June; (2) sandstone; (3) 179.10 m; (4) 10 m a.s.l; (5) coastal garrigue; (6) 23 °C and 10 mm; (7) upper thermomediterranean, lower dry, euoceanic strong; (8) Sardinian, thermomediterranean psammophilous micro-geoseries of coastal dunes (*Cakiletea, Ammophiletea, Crucianellion maritimae, Malcolmietalia, Juniperion turbinatae*)
Teucrium chamaedrys L. subsp. *chamaedrys*	Tcha	CAG 1119a	Pauli longa - Laconi (OR)	39°51'42.31'' N 9° 8'24.87''E	(1) June; (2) dark carbonaceous metapelites; (3) 42644.42 m; (4) 803 m a.s.l; (5) wetland; (6) 20 °C and 50 mm; (7) upper mesomediterranean, lower subhumid, euoceanic weak; (8) central Sardinian, silicicolous, meso-supratemperate series of *Loncomelo pyrenaici-Quercetum ichnusae*
Teucrium flavum L. subsp. *glaucum* (Jord. & Fourr.) Ronniger	Tfsg	CAG 1117a	Nulvi (SS)	40°46'41.51''N 8°44'20.02''E	(1) July; (2) sandstone; (3) 12719.11 m; (4) 513 m a.s.l; (5) garrigue; (6) 22 °C and 20 mm; (7) lower mesomediterranean, lower subhumid, euoceanic weak; (8) central-western Sardinian, silicicolous, mesomediterranean series of *Violo dehnhardtii-Quercetum suberis*
Teucrium marum L.	Tmar	CAG 1115a	Calamosca - Cagliari (CA)	39°11'5.32''N 9° 8'54.79''E	(1) July; (2) sandstone; (3) 130.50 m; (4) 25 m a.s.l; (5) costal garrigue; (6) 24 °C and 10 mm; (7) lower thermomediterranean, lower dry, euoceanic strong; (8) Sardinian thermomediterranean series of *Oleo-Juniperetum turbinatae*
Teucrium massiliense L.	Tmas	CAG 1122a	Loc. Bardacolo - Sorgono (NU)	40° 1'52.21''N 9° 3'56.66''E	(1) June; (2) tonalites; (3) 55326.74 m; (4) 770 m a.s.l; (5) forest; (6) 20 °C and 20 mm; (7) upper mesomediterranean, upper subhumid, semicontinental weak; (8) Sardinian, silicicolous, thermo-mesomediterranean series of *Galio scabri-Quercetum suberis*
Teucrium scordium subsp. *scordioides* (Schreb.) Arcang.	Tsss	CAG 1120a	Pauli longa - Laconi (OR)	39°51'42.31'' N 9° 8'24.87''E	(1) June; (2) dark carbonaceous metapelites; (3) 42644.42 m; (4) 803 m a.s.l; (5) wetland; (6) 20 °C and 50 mm; (7) upper mesomediterranean, lower subhumid, euoceanic weak; (8) central Sardinian, silicicolous, meso-supratemperate series of *Loncomelo pyrenaici-Quercetum ichnusae*

Teucrium scorodonia L.	Tsco	CAG 1118a	Monte Sant'Antonio - Macomer (NU)	40°14'11.01"N 8°40'55.13"E	(1) June; (2) basalt; (3) 18550.93 m; (4) 750 m a.s.l; (5) forest fringes; (6) 19 °C and 20 mm; (7) upper mesomediterranean, upper subhumid, euoceanic weak; (8) central Sardinian, silicicolous, meso-supratemperate series of *Loncomelo pyrenaici-Quercetum ichnusae*
Teucrium subspinosum Pourr. ex Willd.	Tsub	CAG III4a	Marganai Mountain - Iglesias (SU)	39°20'56.20"N 8°34'26.74"E	(1) July; (2) limestone; (3) 10809.04 m; (4) 489 m a.s.l; (5) forest; (6) 22 °C and 10 mm; (7) upper mesomediterranean, upper subhumid, euoceanic weak; (8) central-southern Sardinian, calcicolous, meso-supramediterranean series of *Aceri monspessulani-Quercetum ilicis*

In the last column the following information has been reported: (1) Collection period of plant materials; (2) substratum of collection sites; (3) distance from the sea; (4) altitude; (5) type of habitat; (6) medium temperature and precipitation of the month of collection; (7) bioclimate - isobioclimates; (8) vegetation and vegetation series
